# Decreased total iron binding capacity upon intensive care unit admission predicts red blood cell transfusion in critically ill patients

**DOI:** 10.1371/journal.pone.0210067

**Published:** 2019-01-23

**Authors:** Taro Imaeda, Taka-aki Nakada, Ryuzo Abe, Shigeto Oda

**Affiliations:** Chiba University Graduate School of Medicine, Department of Emergency and Critical Care Medicine, Chiba, Japan; National Yang-Ming University, TAIWAN

## Abstract

**Introduction:**

Red blood cell (RBC) transfusion is associated with poor clinical outcome in critically ill patients. We investigated the predictive value of biomarkers on intensive care units (ICU) admission for RBC transfusion within 28 days.

**Methods:**

Critically ill patients (n = 175) who admitted to our ICU with organ dysfunction and an expected stay of ≥ 48 hours, without hemorrhage, were prospectively studied (derivation cohort, n = 121; validation cohort, n = 54). Serum levels of 12 biomarkers (hemoglobin, creatinine, albumin, interleukin-6 [IL-6], erythropoietin, Fe, total iron binding capacity [TIBC], transferrin, ferritin, transferrin saturation, folate, and vitamin B12) were measured upon ICU admission, days 7, 14, 21 and 28.

**Results:**

Among the 12 biomarkers measured upon ICU admission, levels of hemoglobin, albumin, IL-6, TIBC, transferrin and ferritin were statistically different between transfusion and non-transfusion group. Of 6 biomarkers, TIBC upon ICU admission had the highest area under the curve value (0.835 [95% confidence interval] = 0.765–0.906) for predicting RBC transfusion (cut-off value = 234.5 μg/dL; sensitivity = 0.906, specificity = 0.632). This result was confirmed in validation cohort, whose sensitivity and specificity were 0.888 and 0.694, respectively. Measurement of these biomarkers every seven days revealed that albumin, TIBC and transferrin were statistically different between groups throughout hospitalization until 28 days. In validation cohort, patients in the transfusion group had significantly higher serum hepcidin levels than those in the non-transfusion group (*P* = 0.004). In addition, joint analysis across derivation and validation cohorts revealed that the serum IL-6 levels were higher in the transfusion group (*P* = 0.0014).

**Conclusion:**

Decreased TIBC upon ICU admission has high predictive value for RBC transfusion unrelated to hemorrhage within 28 days.

## Introduction

Hemoglobin is crucial in maintaining adequate oxygen supply during recovery from critical illness [1]. When oxygen supply is insufficient, blood erythropoietin levels increase, and red blood cell production in the bone marrow is thus enhanced. Iron, vitamin B12, and folate facilitate the subsequent erythropoiesis and hemoglobin synthesis [2, 3]. However, more than one-third of patients in intensive care units (ICUs) require red blood cell (RBC) transfusion to maintain a target hemoglobin concentration of 7 g/dL [4, 5]. Moreover, in patients who stay in ICU longer than a week, the proportion of RBC transfusion exceeds 70% [6, 7]. Since anemia and RBC transfusion are associated with worse clinical outcomes in critically ill patients [5, 8], it is important to prevent anemia and the need for RBC transfusion in critically ill patients, which may improve the quality and clinical outcomes of critical care.

Anemia unrelated to hemorrhage in critically ill patients can have a number of causes [9, 10], including insufficient erythropoietin [11] and deficiencies in folate or vitamin B12 [12]. In addition, it may be that inflammatory cytokines, such as interleukin-6 (IL-6), enhance the synthesis of hepcidin, in turn inhibiting iron release from stores, as well as iron entry into the blood. Hepcidin may also decrease serum transferrin levels and total iron binding capacity (TIBC), leading to functional iron deficiency and anemia [13]. However, it is unclear which of these factors contributes significantly to anemia in critically ill patients, which requires RBC transfusion [14].

Thus, we hypothesized that the serum levels of several anemia-related biomarkers upon ICU admission differ between patients who require RBC transfusion unrelated to hemorrhage within 28 days of ICU admission and those who do not. Primarily, we determined the predictive value of these biomarkers for RBC transfusion within 28 days of ICU admission. Furthermore, whether the initial differences in biomarker levels upon ICU admission between patients who require RBC transfusion and those who do not would remain after ICU treatments or not. Thus, we subsequently measured the biomarker levels once a week for 4 weeks after ICU admission and tested for differences. We also measured the quantity of daily blood sampling within 28 days.

## Methods

### Ethical approval

The institutional review board at the Chiba University Graduate School of Medicine approved the current study. Written informed consent was obtained from the patients or their representatives.

### Patients

#### Derivation cohort

The current observational study was conducted prospectively. Consecutive patients (n = 2,101) admitted to the surgical/medical ICU at Chiba University Hospital between January 2016 and March 2017 were screened. Of these, 901 were aged ≥ 20 years and had organ dysfunction according to the Brussels criteria (sequential organ failure assessment [SOFA] score ≥ 2) with an expected ICU stay of ≥ 48 hours or longer. Of these patients, 780 were excluded on the basis of predetermined criteria (bleeding at any time point during hospitalization, chronic hemodialysis, extracorporeal membrane oxygenation, post-operation, trauma, malignancy, during chemotherapy). Ultimately, 121 adult ICU patients were included in derivation cohort.

#### Validation cohort

A further group of consecutive patients (n = 992) admitted to the same ICU between April 2017 and September 2017 were screened for validation cohort. Of these, 54 patients fulfilled the same inclusion/exclusion criteria as above and were included in the validation analysis.

### Collection and definition

The initial blood samples were immediately collected upon ICU admission. Subsequent samples were serially collected at 6 a.m. every 7 days until day 28 during the patients’ hospital stay. A closed blood conservation device (Edwards Lifesciences Corporation, CA, USA) was used to collect the samples from an arterial line. Quantity of blood sampling for clinical examinations were collected according to a predetermined protocol every day up to 28 days. RBC was transfused according to the local protocol to maintain blood hemoglobin concentration at 7 g/dL. Sepsis was defined according to the sepsis-3 definition [15].

### Measurements

Twelve biomarkers were measured in the blood of derivation cohort patients. Of these markers, eight were measured in the clinical laboratories of Chiba University Hospital within 4 hours of collection as routine laboratory measurements. Specifically, these were hemoglobin (Sysmex, Kobe, Japan), albumin, creatinine, Fe, TIBC, ferritin (Denka Seiken Co. Ltd., Tokyo, Japan), IL-6 (Roche Diagnostics, Mannheim, Germany), and transferrin saturation, which is calculated using the Fe and TIBC values. Erythropoietin, folate, vitamin B12 (Beckman Coulter, Tokyo, Japan), and transferrin (Nittobo Medical Co. Ltd., Tokyo, Japan) were measured in an external clinical laboratory (SRL. Inc., Tokyo, Japan). Albumin was measured as nutritional status.

The TIBC appeared to be a significant factor in the derivation cohort. It is known that IL-6 enhances synthesis of hepcidin and that increased hepcidin decreases serum levels of TIBC. Thus, to test for a biological plausibility, we additionally collected plasma and added measurements of hepcidin using a commercial ELISA kit (R&D systems, Minneapolis, USA) in the validation cohort.

### Statistical analysis

The primary outcome variable was RBC transfusion during the 28-day observational period after ICU admission. Differences in baseline characteristics were analyzed using the chi-square test and Mann–Whitney *U* tests for categorical and continuous data, respectively. To estimate the predictive value of the biomarkers for RBC transfusion, the receiver operating characteristic (ROC) curve was derived, and the area under the curve (AUC) was calculated. We compared the ROC curve analysis using DeLong's test. To adjust baseline differences between transfusion and non-transfusion group, we further tested for the association of each biomarker with RBC transfusion using a multivariate logistic regression analysis with adjustments of the baseline differences including age (per year), sex (male or female), patient origin from emergency room (ER or not), sepsis or not and SOFA score (per score). We included patient origin from ER and sepsis to adjust illness and SOFA score to adjust illness severity and looked at the association with transfusion needs. We also examined co-linearity for 12 continuous variables to avoid confounding bias. The Pearson correlation coefficient between the levels of TIBC and transferrin upon ICU admission was analyzed. In validation cohort, we calculated the sensitivity and specificity values of each biomarker using the cut-off values derived from derivation cohort. According to a sample size calculation using the AUC value from derivation cohort (AUC [TIBC] = 0.84, power = 0.95, significance level = 0.05, case:control ratio = 1:1, pROC package for R), the required sample size of the validation cohort was n = 16 per group. *P-*values less than 0.05 were considered statistically significant. A computer software package (GraphPad Prism 7; GraphPad Software Inc., San Diego, CA, USA) was used for all statistical analyses except for calculating the sample size of the validation cohort.

## Results

Of the 2,101 patients screened for derivation cohort, 121 adult ICU patients had (1) organ dysfunction with an expected ICU stay of ≥ 48 hours and (2) no signs of bleeding during the 28-day study period. The median length of the ICU stay was 10 days (interquartile range [IQR] = 5.0–15.5), and the 28-day mortality was 16.5%. Of the 121 patients, 53 (43.8%) received RBC transfusion within 28 days of ICU admission (Table 1). The median RBC transfusion quantity at any one time was 280 mL (IQR = 280–560); the median number of RBC transfusions per patient was three (IQR = 1–5) during the 28-day study period. The median hemoglobin levels before and after RBC transfusion were 6.90 (IQR = 6.55–7.40) and 8.30 (IQR = 7.60–9.05) g/dL, respectively. 37 patients (70%) in the first week, 31 patients (58%) in the second week, 22 patients (42%) in the third week, and 15 patients (28%) in the fourth week from ICU admission were transfused. In the transfusion group, a significantly higher proportion of the patients had been admitted from wards; they also had higher SOFA scores and more sepsis than the non-transfusion group (Table 1).

**Table 1:** Baseline characteristics and outcomes (derivation cohort).

	Transfusion^a^ (n = 53)	Non-transfusion (n = 68)	*P*-value^b^
Age, year	69 (61–75)^c^	65 (58–75)	.34
Male, n (%)	39 (74)	51 (75)	.86
BMI, kg/m^2^	23.2 (20.0–26.5)	23.2 (21.1–26.2)	.75
From ER/wards, n	13/40	37/31	.0009
SOFA score	13 (9–16)	8 (6–10)	< .0001
Sepsis, n (%)	37 (70)	27 (40)	.0010
Diagnosis category^d^			
Circulatory, n (%)	5 (9)	23 (34)	.0020
Respiratory, n (%)	18 (34)	22 (32)	1.0
Digestive, n (%)	12 (23)	6 (9)	.0042
Genitourinary, n (%)	5 (9)	4 (6)	.50
Nervous, n (%)	0 (0)	4 (6)	.13
Other, n (%)	13 (25)	9 (13)	.15
Data on ICU admission			
Hemoglobin (g/dL)	9.6 (8.3–13.0)	12.6 (11.1–14.2)	< .0001
Creatinine (mg/dL)	1.39 (0.95–2.55)	1.00 (0.74–1.80)	.060
Albumin (g/dL)	2.4 (1.9–2.9)	3.2 (2.5–3.5)	< .0001
IL-6 (pg/mL)	721 (267–4870)	140 (54–409)	.0001
Erythropoietin (mIU/mL)	36.3 (13.0–67.8)	26.1 (10.5–50.2)	.20
Fe (μg/dL)	42.0 (24.0–80.0)	52.0 (34.0–75.0)	.37
TIBC (μg/dL)	165 (110–208)	250 (200–305)	< .0001
Transferrin (mg/dL)	109 (78–147)	173 (130–210)	< .0001
Ferritin (ng/mL)	870 (367–2678)	301 (120–577)	< .0001
TSAT (%)	27.8 (12.7–42.9)	19.1 (12.7–30.5)	.13
Folate (ng/mL)	6.7 (4.9–10.2)	7.9 (4.7–10.5)	.37
Vitamin B12 (pg/mL)	902 (552–2675)	676 (290–1278)	.064
RBC transfusion volume at any one time, mL	280 (280–560)	–	–
Number of RBC transfusions per patient, n	3 (1–5)	–	–
Outcomes			
Cumulative amount of exsanguination^e^, mL	445.4 (377.0–568.8)	288.0 (173.8–363.9)	< .0001
Length of ICU stay, days	14 (10–24)	8 (4–10)	< .0001
Length of hospital stay, days	57 (23–87)	29 (17–46)	< .0030
28-day mortality, n (%)	11 (21)	9 (13)	.26

Abbreviations: BMI, body mass index; ER, emergency room; IL-6, Interleukin-6; SOFA, sequential organ failure assessment; TIBC, total iron binding capacity; TSAT, Transferrin saturation; RBC, red blood cell

^a^ Transfusion indicates red blood cell transfusion.

^b^ *P*-values were calculated using chi-square test or Mann–Whitney *U* tests.

^c^ Data are expressed as median values (interquartile range) for continuous variables.

^d^ Diagnosis category is formed according to ICD-11.

^e^ Data are the quantity of blood sampling during the 28 days after ICU admission.

**Fig 1:**
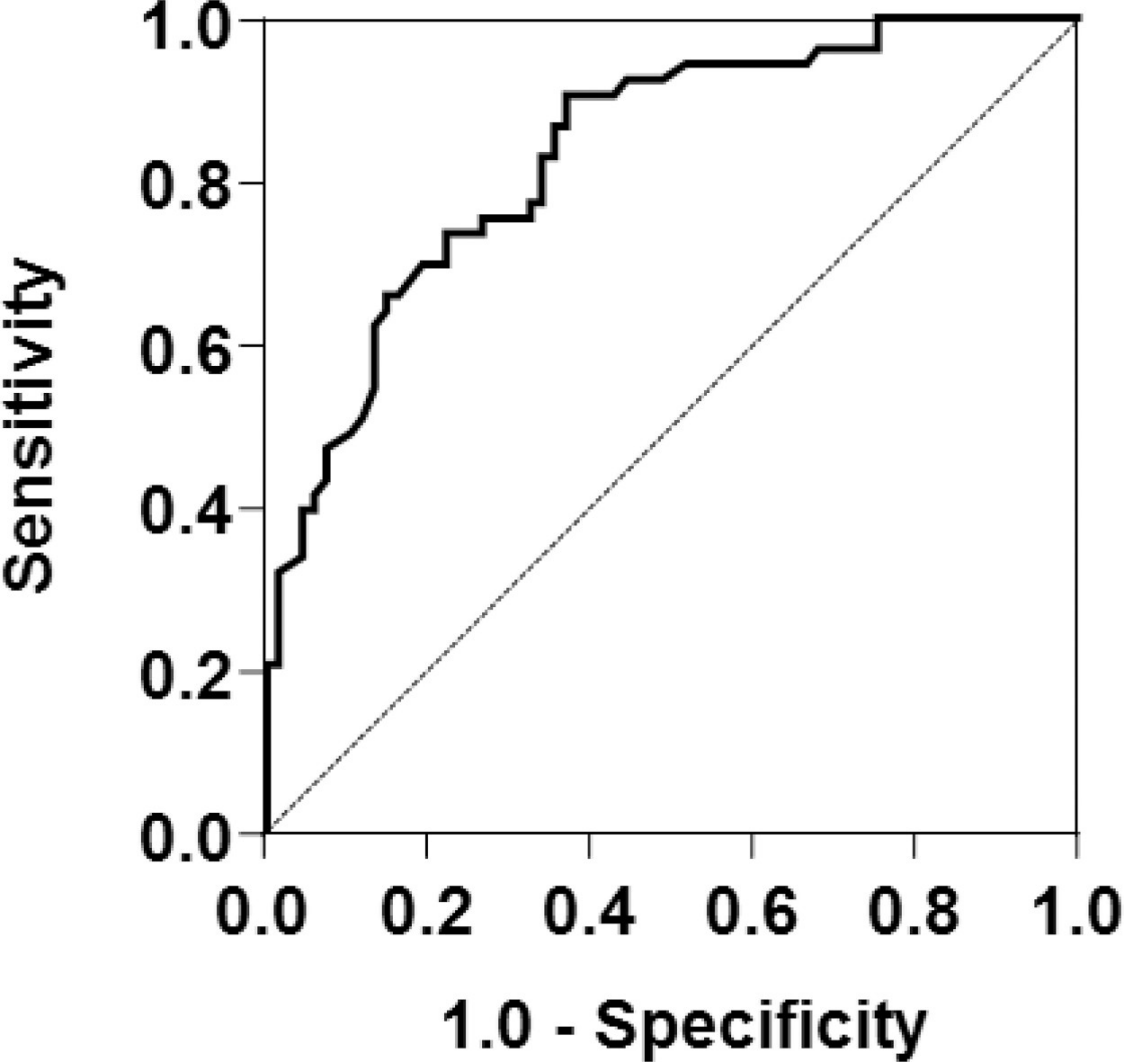
Receiver-operating characteristic curve analysis of whether total iron binding capacity (TIBC) upon ICU admission can predict RBC transfusion within 28 days.

With regards to the 12 biomarkers measured upon ICU admission, patients in the transfusion group had significantly lower levels of blood hemoglobin, albumin, TIBC, and transferrin, as well as significantly higher levels of IL-6 and ferritin than the non-transfusion group (Table 1). In the ROC analysis of these 6 biomarkers upon ICU admission that showed a significant difference, TIBC had the highest AUC value (0.835, 95% confidence interval [CI] = 0.765–0.906; Table 2; Fig 1). The AUC of TIBC was significantly higher than that of hemoglobin, albumin or IL-6 (hemoglobin *P* = 0.011, albumin *P* = 0.011, IL-6 *P* = 0.026, Transferrin *P* = 0.45, Ferritin *P* = 0.11). In the multivariate logistic regression analysis to adjust the baseline differences for age, male gender, patient origin from ER, SOFA score, and sepsis (Table 1), decreased TIBC and transferrin upon ICU admission remained highly associated with RBC transfusion within 28 days of ICU admission (*P* < 0.001; Table 2) (ROC curve; S1 Fig).

The area under the curve (AUC) was 0.835 (95% confidence interval = 0.765–0.906).

**Table 2:** Receiver operating characteristic curve analysis for predicting RBC transfusion within 28 days.

				Univariate	Multivariate^b^
	AUC (95% CI)	Cut-off value^a^	Sensitivity, specificity	*P*-value (OR [95%CI])	*P*-value^c^ (OR [95%CI])
Hemoglobin	0.709 (0.610–0.809)	10.7 g/dL	0.809, 0.623	< .0001 (0.75 [0.65–0.87])	.0003 (0.70 [0.57–0.84])
Albumin	0.720 (0.624–0.816)	2.85 g/dL	0.623, 0.740	< .0001 (0.34 [0.18–0.59])	.0005 (0.29 [0.14–0.56])
IL-6	0.639 (0.524–0.754)	248.3 pg/mL	0.591, 0.745	.0672 (1.00 [1.00–1.00])	.1908 (1.00 [1.00–1.00])
TIBC	0.835 (0.765–0.906)	234.5 μg/dL	0.906, 0.632	< .0001 (0.98 [0.97–0.99])	< .0001 (0.97 [0.96–0.98])
Transferrin	0.815 (0.734–0.895)	116.5 mg/dL	0.842, 0.638	< .0001 (0.97 [0.96–0.98])	< .0001 (0.97 [0.96–0.99])
Ferritin	0.758 (0.672–0.843)	590.6 ng/mL	0.765, 0.660	< .0001 (1.00 [1.00–1.00])	.0066 (1.00 [1.00–1.00])

Abbreviations: RBC, red blood cell; AUC, area under receiver-operating characteristic curve; CI, confidence interval; OR, odds ratio; IL-6, Interleukin-6; TIBC, total iron binding capacity

^a^ The Youden index was used to determine cut-off values.

^b^ Adjusted for age (per year), male gender, ER, SOFA score (per score), and sepsis.

^c^ *P-*values were calculated using logistic regression.

Then, we examined co-linearity for continuous variables to avoid confounding bias. As a result, among all combinations of 12 factors, only relationship between TIBC and transferrin demonstrated significantly high correlation to each other (R value > 0.7), while albumin-hemoglobin, albumin-TIBC, albumin-transferrin and Fe-TSAT showed moderate correlation of R value > 0.5.

Transferrin levels were significantly correlated with TIBC (R value = 0.90, *P* < 0.0001; S2 Fig). TIBC is routinely measured in in-hospital clinical laboratories, while transferrin is measured in external clinical laboratories that have higher costs and take a longer time to confirm their results. For this reason, we focused on TIBC in the replication cohort.

The predictive value of TIBC upon ICU admission was analyzed in validation cohort (baseline characteristics in S1 Table). Using the cut-off value calculated from derivation cohort (234.5 μg/dL; Table 2), the sensitivity and specificity of TIBC for predicting RBC transfusion within 28 days of ICU admission were 0.888 and 0.694, respectively, in validation cohort (ROC curve; S1 Fig). Joint analysis across derivation and validation cohorts (n = 175) using ROC curve analysis yielded the same conclusion for predicting RBC transfusion within 28 days (AUC [95% CI] = 0.837 [0.777–0.897]; cut-off value = 228 μg/dL; sensitivity = 0.873; specificity = 0.689; OR [95% CI] = 0.98 [0.97–0.99]). To test for a biological plausibility of decreased TIBC levels, we evaluated serum levels of hepcidin and IL-6 in validation cohort. Patients in the transfusion group had significantly higher serum levels of hepcidin (169.2 ng/mL [98.1–224.9 ng/mL]) than those in the non-transfusion group (62.2 ng/mL [26.5–111.7], *P* = 0.004; S2 Table). There was a non-significant trend towards higher IL-6 levels in the transfusion group (225.1 pg/mL [126.4–2671.0 pg/mL]) than in the non-transfusion group (105.2 pg/mL [58.6–451.6 pg/mL], *P* = 0.06; S2 Table); however, joint analysis across derivation and validation cohorts revealed that the serum IL-6 levels in the transfusion group (546.7 pg/mL [157.6–4957.0 pg/mL]) were higher than those in the non-transfusion group (161.2 pg/mL [50.3–759.8 pg/mL], *P* = 0.0014).

Next, we tested for differences in serum levels of the biomarkers during the 28 days after ICU admission. Throughout the observational period, patients in the transfusion group had significantly lower levels of serum albumin, TIBC, and transferrin and had higher level of ferritin than patients in the non-transfusion group (*P* < 0.05; Table 3; S3 Table).

**Table 3:** Serum levels of three biomarkers and cumulative amount of exsanguination during the 28-day period after ICU admission.

	Transfusion^a^	Non-transfusion	*P*-value^b^
Albumin, g/dL			
Day 1 (n = 53, n = 68)^c^	2.3 (1.9–2.8)^d^	3.2 (2.5–3.6)	< .0001
Day 7 (47, 65)	2.0 (1.7–2.3)	2.4 (2.0–2.9)	.0010
Day 14 (37, 63)	2.1 (1.8–2.5)	2.7 (2.1–3.2)	.0003
Day 21 (27, 54)	2.2 (1.7–2.6)	3.1 (2.4–3.6)	< .0001
Day 28 (15, 44)	2.2 (1.7–2.9)	3.2 (2.5–3.7)	.0006
TIBC, μg/dL			
Day 1 (53, 68)	163.0 (110.0–218.0)	261.5 (199.5–314.0)	< .0001
Day 7 (47, 65)	145.0 (117.0–177.0)	208.0 (165.0–254.0)	< .0001
Day 14 (37, 63)	167.0 (143.0–205.0)	237.0 (192.0–286.0)	.0002
Day 21 (27, 54)	165.0 (136.0–203.5)	265.5 (202.3–303.3)	< .0001
Day 28 (15, 44)	159.0 (128.0–225.5)	288.0 (235.0–309.0)	.0014
Transferrin, mg/dL			
Day 1 (53, 68)	109.0 (78.0–147.0)	177.0 (129.0–211.0)	< .0001
Day 7 (47, 65)	106.0 (91.0–128.0)	154.0 (122.5–182.0)	< .0001
Day 14 (37, 63)	122.0 (100.0–141.5)	175.0 (148.5–236.5)	< .0001
Day 21 (27, 54)	122.5 (95.3–148.3)	206.0 (166.3–244.5)	< .0001
Day 28 (15, 44)	114.0 (89.8–139.3)	225.0 (200.5–243.0)	< .0001
Cumulative amount of exsanguination, mL			
Day 1—Day 7 (53, 68)^e^	240.5 (210.0–289.5)	182.8 (137.5–243.0)	< .0001
Day 8—Day 14 (47, 65)	170.0 (122.0–214.0)	53.8 (24.0–96.3)	< .0001
Day 15—Day 21 (37, 63)	80.0 (30.0–166.0)	20.5 (0–37.0)	< .0001
Day 22—Day 28 (27, 54)	50.0 (0–110.0)	0 (0–26.0)	< .0001

Abbreviation: TIBC, Total iron binding capacity

^a^ Transfusion indicates red blood cell transfusion.

^b^ *P*-values were calculated using Mann–Whitney *U* test.

^c^ Total number of patients (number of transfusion, non-transfusion group) who were

measrued serum levels of albumin, TIBC and transferin was 121 (53, 68) on Day 1, 112 (47

65) on Day 7, 100 (37, 63) on Day 14, 81 (27, 54) on Day 21, 59 (15, 44) on Day 28.

^d^ Data are expressed as median values (interquartile range) for continuous variables.

^e^ Total number of patients (number of transfusion, non-transfusion group) who were measured the quantity of blood sampling every week was 121 (53, 68) at the first week, 112 (47, 65) at the second week, 100 (37, 63) at the third week, 81 (27, 54) at the fourth week.

Finally, we further tested for a difference of the comparison of the quantity of blood sampling among the two groups. Patients in the transfusion group had increased quantity of blood collection compared to the non-transfusion group (average quantity of daily blood sampling, transfusion group 21.7 ± 10.4 mL/day; non-transfusion group, 15.1 ± 7.2 mL/day, *P* = 0.0003). In ROC analysis of quantity of blood sampling on RBC transfusion within 28 days, the AUC value was 0.723 (95% CI = 0.627–0.818).

## Discussion

In the present study, which investigated 12 anemia-related biomarkers in critically ill patients, serum TIBC upon ICU admission had the highest predictive value for RBC transfusion unrelated to hemorrhage within 28 days of ICU admission. The TIBC value had a significantly higher predictive value compared to the hemoglobin or albumin. In the multivariate analysis, decreased serum TIBC upon ICU admission was an independent risk factor for RBC transfusion. Patients in the transfusion group had higher levels of hepcidin and IL-6 upon ICU admission than those in the non-transfusion group.

The TIBC test measures the maximum amount of iron that serum (or plasma) can bind [16]. Transferrin is the major carrier of iron, and the TIBC test is used as an indirect test of transferrin levels. However, the TIBC test may be inaccurate in this regard, because other proteins, such as albumin, bind to iron, and thus the test may overestimate transferrin levels [17]. Conversely, the TIBC test is cheaper than direct transferrin measurement, and it is therefore more widely used [18]. To our knowledge, no previous studies have reported that TIBC upon ICU admission has a high predictive value for anemia unrelated to hemorrhage in critically ill patients. Of course, TIBC is not currently measured routinely in critically ill patients; however, TIBC is commonly measured for the examination in patient with anemia. In addition, levels of hemoglobin or albumin is more easily obtained in critically ill patients; however the AUC of hemoglobin or albumin was lower than that of TIBC upon ICU admission in the ROC analysis for predicting RBC transfusion within 28 days (hemoglobin 0.709; albumin 0.720; TIBC 0.835). TIBC upon ICU admission had the highest AUC value in anemia-related biomarkers. Therefore, Measurement of TIBC upon ICU admission may become the first step towards developing the approach such as hepcidin-targeting therapy or reduction of phlebotomy-associated blood loss to preventing anemia or RBC transfusion in critically ill patients.

Inflammation is a key component in the complex pathophysiology of critically ill patients, and it can also be a cause of anemia. In this study, patients in the transfusion group had higher levels of inflammatory biomarkers, such as IL-6 and ferritin, as well as lower TIBC and transferrin levels as biomarkers of anemia, upon ICU admission than those in the non-transfusion group. Furthermore, increased level of hepcidin was observed in the transfusion group. It is reported that inflammatory cytokines such as IL-6 enhanced synthesis of hepcidin, which is a principal regulator of iron absorption [19, 20, 21]. Serum levels of transferrin and TIBC are reportedly decreased in response to prevented incorporation of iron into erythroid precursors by the effect of increased hepcidin [22, 23]. That is the reason why hepcidin-targeting therapy is expected to be a new treatment for critically ill patients [24, 25]. We found that lower TIBC upon ICU admission is a risk factor for subsequent transfusion, and that it is potentially associated with increased hepcidin. Therefore, decreased TIBC may be a good entry criterion for the hepcidin-target therapy.

Another feasible approach to prevent anemia and RBC transfusion in critically ill patients may be reduction of phlebotomy-associated blood loss. Previous studies showed amounts of daily blood sampling in critically ill patients ranged from 23 to 40 mL/day [26]. In accord with these, in the current study, the amount ranged from 15.1 ± 7.2 mL/day (non-transfusion group) to 21.7 ± 10.4 mL/day (transfusion group). Since small volume pediatric sampling bottles was suggested as a potential approach to reduce phlebotomy-associated blood loss [27] and the transfusion group had increased quantity of blood collection in the current study, the use of small volume pediatric sampling bottles may effectively reduce RBC transfusion in patients who had decreased TIBC upon ICU admission. Further studies on either hepcidin-target therapy or small volume pediatric sampling bottles in patients with decreased TIBC upon ICU admission would strength the clinical importance of the study result and may change clinical practice.

In response to decreased tissue oxygenation, erythropoietin produced by the kidneys stimulates erythroid progenitor cells in the bone marrow and stimulates RBC production. In a previous study, serum levels of erythropoietin did not differ significantly between patients with critical illness and those without [12, 28]. Accordingly, in the present study, serum levels of erythropoietin did not differ significantly between critically ill patients in the transfusion group and those in the non-transfusion group, indicating that serum levels of erythropoietin upon ICU admission are not predictive of subsequent transfusion in critically ill patients. Moreover, we found no difference in serum levels of creatinine, which is a biomarker of kidney function, between the two groups. Since the kidney produces and secretes erythropoietin, this similar kidney function may be connected to the similar erythropoietin levels. In addition, in a randomized controlled trial, erythropoietin therapy in critically ill patients failed to reduce the number of RBC transfusions, and it increased thrombotic events [11]. Thus, this therapy is not recommended for critically ill patients [29], and it is unlikely as a future therapeutic target in critically ill patients.

Iron, folate, and vitamin B12 may be deficient in critically ill patients due to insufficient nutrition. This may contribute to anemia. Indeed, iron deficiency is common in critically ill patients, affecting 30%–40% of them [13, 30]. However, iron, folate, or vitamin B12 deficiency-induced anemia is not common in critically ill patients, affecting only 9%, 2%, and 2% of patients, respectively [12]. In keeping with these results, we found that the median serum levels of iron were lower than normal in the present study, while folate and vitamin B12 levels were within the normal range. Furthermore, serum levels of these three biomarkers did not differ between critically ill patients in the transfusion and non-transfusion groups. Thus, it is unlikely that these biomarkers can be used upon ICU admission to predict subsequent transfusion. In a randomized control trial, intravenous iron administration failed to reduce the number of RBC transfusions among anemic patients in an ICU [31], and excessive iron increases a patient’s susceptibility to infection [32]. Thus, iron is unlikely to be a therapeutic target in critically ill patients.

The present study had several limitations. First, although we found that TIBC upon ICU admission has a significant predictive value for RBC transfusion in both the derivation and validation cohorts, the sample size was not large. Second, to identify specific factors of critical care-related anemia, we limited subjects with SOFA score ≥ 2 and expected ICU stay of ≥ 48 hours and excluded patients who had any potential confounding factors such as bleeding, chronic hemodialysis, extracorporeal membrane oxygenation, post-operation, trauma, malignancy and during chemotherapy, which resulted in 5% enrollment rate of the ICU admission and may limit the generalizability. Third, this study was performed in a single center. Lastly, these anemia-related biomarkers were evaluated only at fixed timing from ICU admission, i.e. days 1, 7, 14, 21 and 28, which was independent of timing of RBC transfusion. There is a possibility that levels of these biomarkers were affected by RBC transfusion. Further studies in multiple centers will strengthen the results of the present study.

## Conclusion

Decreased TIBC upon ICU admission has highest predictive value for subsequent RBC transfusion within 28 days of ICU admission among 12 anemia-related biomarkers. The TIBC value had a significantly higher predictive value compared to the hemoglobin or albumin. Further investigation to develop a strategy for reducing RBC transfusion in high-risk patients is warranted.

## Supporting information

S1 Fig Receiver-operating characteristic curve analysis of whether total iron binding capacity (TIBC) upon ICU admission can predict RBC transfusion within 28 days. The area under the curve (AUC) was 0.835 (95% confidence interval [CI] = 0.765–0.906) in the univariate analysis of derivation cohort, 0.877 (95% CI = 0.818–0.937) in the multivariate logistic regression analysis of derivation cohort, and 0.826 (95% CI = 0.700–0.952) in the univariate analysis of validation cohort.
(TIFF)

S2 Fig A scatter plot comparing total iron binding capacity (TIBC) and transferrin levels n = 121, transferrin (mg/dL) = 0.716762 × TIBC (μg/dL)– 6.521468, R value = 0.9022, *P* < 0.0001. (TIF)

S1 Table Baseline characteristics in validation cohort. (DOCX)

S2 Table Serum levels of hepcidin and interleukin-6 upon ICU admission in validation cohort. (DOCX)

S3 Table Serum levels of 9 biomarkers during the 28-day period after ICU admission. (DOCX)
