# Supplementary figures and images for: Decreased total iron binding capacity upon intensive care unit admission predicts red blood cell transfusion in critically ill patients
Source: PLoS One. 2019 Jan 23;14(1):e0210067. doi: 10.1371/journal.pone.0210067 (PMC6343884; doi:10.1371/journal.pone.0210067)

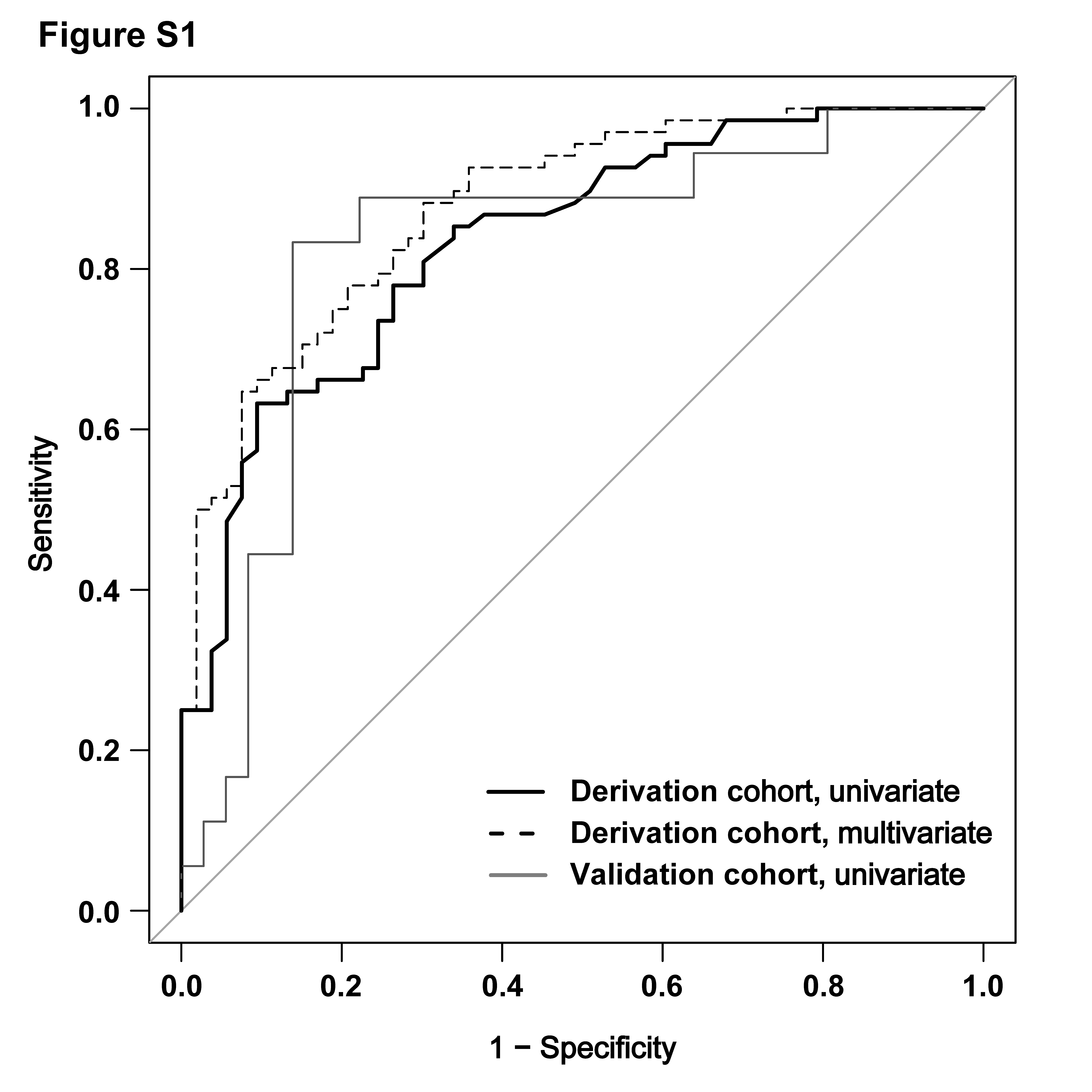

Supplement: S1 Fig — The area under the curve (AUC) was 0.835 (95% confidence interval [CI] = 0.765–0.906) in the univariate analysis of derivation cohort, 0.877 (95% CI = 0.818–0.937) in the multivariate logistic regression analysis of derivation cohort, and 0.826 (95% CI = 0.700–0.952) in the univariate analysis of validation cohort. (TIFF) [file pone.0210067.s001.tiff]

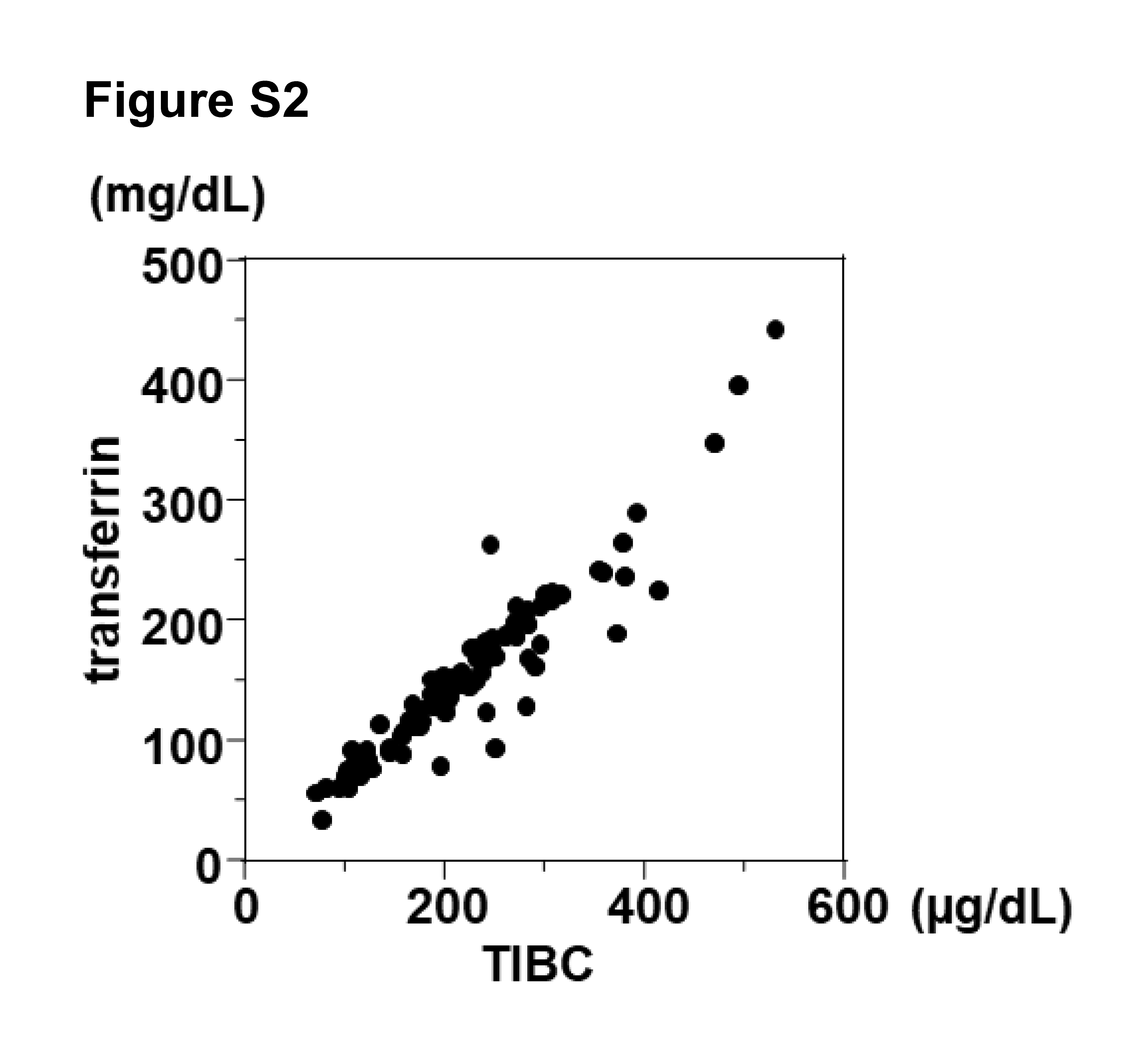

Supplement: S2 Fig — (TIF) [file pone.0210067.s002.tif]
