# Supplementary material for: Decreased total iron binding capacity upon intensive care unit admission predicts red blood cell transfusion in critically ill patients
Source: PLoS One. 2019 Jan 23;14(1):e0210067. doi: 10.1371/journal.pone.0210067 (PMC6343884; doi:10.1371/journal.pone.0210067)
Supplement: S1 Table — (DOCX) [file pone.0210067.s003.docx]

**Table S1. Baseline characteristics in validation cohort**

|  | Transfusion^a^ (n = 18) | Non-transfusion (n = 36) | *P*-value^b^ |
| --- | --- | --- | --- |
| Age, year | 70 (59–75)^c^ | 67 (43–74) | .33 |
| Male, n (%) | 9 (50) | 21 (59) | .58 |
| BMI, kg/m^2^ | 23.3 (19.7–28.1) | 24.0 (20.2–26.6) | .96 |
| From ER/wards, n | 5/13 | 16/20 | .37 |
| SOFA score | 9 (7–16) | 8 (6–10) | .055 |
| Sepsis, n (%) | 12 (67) | 15 (42) | .15 |
| Diagnosis category^d^ |  |  |  |
| Circulatory, n (%) | 4 (22) | 7 (19) | .81 |
| Respiratory, n (%) | 3 (17) | 9 (25) | .49 |
| Digestive, n (%) | 4 (22) | 7 (19) | .81 |
| Genitourinary, n (%) | 2 (11) | 2 (6) | .46 |
| Nervous, n (%) | 1 (6) | 2 (6) | 1.0 |
| Other, n (%) | 4 (22) | 9 (25) | .82 |

Abbreviations: BMI, body mass index; ER, emergency room; SOFA, sequential organ failure assessment

^a^ Transfusion indicates red blood cell transfusion.

^b^ *P*-values were calculated using the chi-square test or Mann–Whitney *U* test.

^c^ Data are expressed as median values (interquartile range) for continuous variables.

^d^ Diagnosis category is formed according to ICD-11.
