# Supplementary material for: Decreased total iron binding capacity upon intensive care unit admission predicts red blood cell transfusion in critically ill patients
Source: PLoS One. 2019 Jan 23;14(1):e0210067. doi: 10.1371/journal.pone.0210067 (PMC6343884; doi:10.1371/journal.pone.0210067)
Supplement: S2 Table — (DOCX) [file pone.0210067.s004.docx]

**Table S2. Serum levels of hepcidin and interleukin-6 upon ICU admission in validation cohort**

|  | Transfusion^a^ (n = 18) | Non-transfusion (n = 36) | *P*-value^b^ |
| --- | --- | --- | --- |
| Hepcidin, ng/mL | 169.2 (98.1–224.9)^c^ | 62.2 (26.5–111.7) | .004 |
| Interleukin-6, pg/mL | 225.1 (126.4–2671.0) | 105.2 (58.6–451.6) | .06 |

^a^Transfusion indicates red blood cell transfusion.

^b^*P*-values were calculated using chi-square test or Mann–Whitney *U* tests.

^c^Data are expressed as median values (interquartile range) for continuous variables.
