# Supplementary material for: Decreased total iron binding capacity upon intensive care unit admission predicts red blood cell transfusion in critically ill patients
Source: PLoS One. 2019 Jan 23;14(1):e0210067. doi: 10.1371/journal.pone.0210067 (PMC6343884; doi:10.1371/journal.pone.0210067)
Supplement: S3 Table — (DOCX) [file pone.0210067.s005.docx]

**Table S3. Serum levels of 9 biomarkers during the 28-day period after ICU admission**

|  | Transfusion^a^ (n = 53) | Non-transfusion (n = 68) | *P*-value^b^ |
| --- | --- | --- | --- |
| Hemoglobin, g/dL |  |  |  |
| Day 1 | 9.6 (8.3–13.0)^c^ | 12.6 (11.1–14.2) | <.0001 |
| Day 7 | 8.9 (7.8–9.9) | 10.7 (9.2–12.3) | <.0001 |
| Day 14 | 8.7 (7.8–9.4) | 10.8 (9.4–11.9) | <.0001 |
| Day 21 | 8.5 (7.8–9.4) | 11.1 (9.5–11.9) | <.0001 |
| Day 28 | 8.6 (8.0–9.6) | 11.2 (9.6–12.9) | <.0001 |
| Creatinine, mg/dL |  |  |  |
| Day 1 | 1.39 (0.95–2.55) | 1.00 (0.74–1.80) | .0607 |
| Day 7 | 1.00 (0.69–1.66) | 0.72 (0.56–1.20) | .0083 |
| Day 14 | 1.07 (0.67–2.14) | 0.70 (0.56–0.97) | .0044 |
| Day 21 | 0.98 (0.62–1.57) | 0.73 (0.54–0.98) | .0241 |
| Day 28 | 0.80 (0.57–1.84) | 0.64 (0.50–0.89) | .0985 |
| IL-6, pg/mL |  |  |  |
| Day 1 | 707.9 (195.6–5384.0) | 196.2 (43.8–2479.8) | .0229 |
| Day 7 | 122.6 (14.3–220.7) | 59.1 (32.9–140.4) | .6685 |
| Day 14 | 40.3 (18.7–127.2) | 18.1 (6.4–26.3) | .1101 |
| Day 21 | - | - | - |
| Day 28 | - | - | - |
| Erythropoietin, mIU/mL |  |  |  |
| Day 1 | 37.5 (12.9–73.5) | 22.6 (10.0–49.7) | .0988 |
| Day 7 | 19.1 (12.6–51.4) | 18.1 (9.3–33.1) | .1971 |
| Day 14 | 23.4 (8.7–58.8) | 14.3 (9.1–32.0) | .1047 |
| Day 21 | 25.1 (9.9–98.2) | 12.5 (7.8–20.1) | .0332 |
| Day 28 | 25.1 (13.7–53.7) | 11.1 (6.8–23.9) | .0045 |
| Fe, µg/dL |  |  |  |
| Day 1 | 41.5 (22.5–77.0) | 52.0 (34.0–76.0) | .2268 |
| Day 7 | 52.0 (25.8–106.3) | 44.5 (31.3–66.3) | .3131 |
| Day 14 | 59.0 (37.5–109.5) | 51.5 (33.0–74.8) | .1108 |
| Day 21 | 54.0 (31.0–91.0) | 50.5 (31.3–81.5) | .7671 |
| Day 28 | 67.5 (44.8–107.8) | 55.0 (32.0–83.0) | .2060 |
| Ferritin, ng/mL |  |  |  |
| Day 1 | 870.0 (366.8–2678.0) | 280.0 (121.5–608.1) | <.0001 |
| Day 7 | 642.2 (327.3–1292.3) | 342.0 (189.8–579.3) | <.0001 |
| Day 14 | 632.0 (392.5–1289.0) | 316.1 (188.4–511.3) | .0003 |
| Day 21 | 632.5 (357.0–1144.3) | 274.1 (157.5–455.6) | .0004 |
| Day 28 | 591.6 (365.7–1323.8) | 241.1 (140.5–486.9) | .0062 |
| TSAT, % |  |  |  |
| Day 1 | 29.0 (12.6–44.0) | 19.4 (12.6–31.2) | .1034 |
| Day 7 | 32.7 (17.0–73.8) | 21.2 (14.6–34.7) | .0042 |
| Day 14 | 34.6 (22.6–62.5) | 23.0 (13.5–28.5) | .0002 |
| Day 21 | 31.0 (16.1–43.3) | 19.6 (12.2–28.0) | .0215 |
| Day 28 | 38.5 (26.8–50.0) | 19.0 (14.3–26.1) | .0029 |

|  | Transfusion^a^ (n = 53) | Non-transfusion (n = 68) | *P*-value^b^ |
| --- | --- | --- | --- |
| Folate, ng/mL |  |  |  |
| Day 1 | 6.9 (4.9–10.5) | 8.9 (4.3–11.5) | .4000 |
| Day 7 | 6.9 (4.8–9.0) | 5.9 (4.0–8.0) | .1255 |
| Day 14 | 7.7 (5.9–10.7) | 5.9 (4.9–8.1) | .0088 |
| Day 21 | 8.6 (6.6–12.4) | 5.7 (4.3–10.4) | .0677 |
| Day 28 | 10.8 (6.6–13.9) | 8.3 (5.7–11.5) | .2701 |
| Vitamin B12, pg/mL |  |  |  |
| Day 1 | 914.0 (554.0–3400.0) | 633.0 (273.3–1570.0) | .0977 |
| Day 7 | 1720.0 (855.0–3810.0) | 789.0 (385.0–1290.0) | .0003 |
| Day 14 | 1280.0 (647.0–2740.0) | 703.0 (429.0–997.5) | .0059 |
| Day 21 | 845.5 (508.0–1877.5) | 696.0 (439.5–1000.5) | .2654 |
| Day 28 | 749.0 (475.5–1063.0) | 579.0 (465.0–739.0) | .2547 |

Abbreviations: IL-6, Interleukin-6; TSAT, Transferrin saturation

Total number of patients (number of transfusion, non-transfusion group) who were measrued serum levels of albumin, TIBC and transferin was 121 (53, 68) on Day 1, 112 (47, 65) on Day 7, 100 (37, 63) on Day 14, 81 (27, 54) on Day 21, 59 (15, 44) on Day 28.

^a^Transfusion indicates red blood cell transfusion.

^b^*P*-values were calculated using the Mann–Whitney *U* test.

^c^Data are expressed as median values (interquartile range) for continuous variables.
